# Glucagon‐Like Peptide‐1 Receptor Agonists and Incident Major Adverse Liver Outcomes in People With Type 2 Diabetes and Metabolic Dysfunction‐Associated Steatotic Liver Disease

**DOI:** 10.1111/dom.71023

**Published:** 2026-06-22

**Authors:** Gregor A. Maier, Ramon A. Hofmann, Michael Roden, Wolfgang Rathmann, Oliver Kuss

**Affiliations:** ^1^ Institute for Biometrics and Epidemiology German Diabetes Center, Leibniz Center for Diabetes Research at Heinrich Heine University Düsseldorf Germany; ^2^ German Center for Diabetes Research, Partner Düsseldorf München‐Neuherberg Germany; ^3^ Division of Endocrinology and Diabetology, Medical Faculty and University Hospital Heinrich Heine University, University Hospital Düsseldorf Germany; ^4^ Institute for Clinical Diabetology German Diabetes Center, Leibniz Center for Diabetes Research at Heinrich Heine University Düsseldorf Germany; ^5^ Centre for Health and Society, Medical Faculty and University Hospital Düsseldorf Heinrich Heine University Düsseldorf Germany

**Keywords:** antidiabetic drug, effectiveness, fatty liver disease, observational study, pharmaco‐epidemiology

## Abstract

**Aims:**

Treatment options for metabolic dysfunction‐associated steatotic liver disease (MASLD) are limited. While glucagon‐like peptide‐1 receptor agonists (GLP‐1 RA) and sodium‐glucose cotransporter‐2 (SGLT‐2) inhibitors improve cardiovascular outcomes, comparative effectiveness on liver‐related outcomes remains unclear. This study compared the effectiveness of GLP‐1 RAs versus SGLT‐2 inhibitors on major adverse liver outcomes (MALO), liver cirrhosis and all‐cause mortality in people with MASLD and type 2 diabetes.

**Materials and Methods:**

This active comparator, new‐user cohort study used claims data from Germany (2005–2024), including 45 256 people with MASLD and type 2 diabetes. New users of GLP‐1 RAs (*n* = 9993) and SGLT‐2 inhibitors (*n* = 35 263) were weighted using matching weights. The primary outcome was MALO, while secondary outcomes comprised individual MALO components (decompensation events, liver transplantation, hepatocellular carcinoma (HCC)), liver cirrhosis and all‐cause mortality. Hazard ratios (HR) with 95% confidence intervals (CI) were estimated with weighted Cox proportional hazard models.

**Results:**

Over a median 4.3‐year follow‐up, new users of GLP‐1 RAs had a lower hazard of MALO (HR 0.91, 95% CI 0.78–1.07). This association appeared stronger using an on‐treatment approach (HR 0.78, 95% CI 0.58–1.03) and when restricting to hospital diagnoses in primary position (HR 0.77, 95% CI 0.56–1.07). Benefits were also observed for liver cirrhosis (HR 0.88), decompensation events (HR 0.91), HCC (HR 0.76), but not all‐cause mortality (HR 1.04).

**Conclusions:**

GLP‐1 RAs were associated with a potentially lower hazard for incident MALO and liver cirrhosis compared with SGLT‐2 inhibitors in people with MASLD and type 2 diabetes, suggesting a possible therapeutic advantage for liver‐specific outcomes in this population.

## 1 Introduction

Metabolic dysfunction‐associated steatotic liver disease (MASLD) affects approximately 65% of people with type 2 diabetes, with metabolic dysfunction‐associated steatohepatitis (MASH) present in about 32% of them [1]. MASH, the progressive inflammatory form, frequently associates with fibrosis, which with increasing stage (F0 to F4) raises the risk of end‐stage liver disease [2] and mortality [3], particularly in people with type 2 diabetes [4]. Resmetirom has been approved in the US for MASH with F2–3 fibrosis. It improved histological outcomes but had no effect on body weight or glycemic control [5]. Current guidelines and consensus reports, however, recommend weight loss in people with overweight or obesity to improve MASLD by lifestyle management [6, 7]. Moreover, bariatric (metabolic) surgery has been shown to be effective in improving MASLD histology and outcomes [8]. In August 2025, semaglutide 2.4 mg, a glucagon‐like peptide‐1 receptor agonist (GLP‐1 RA), was approved in the US for MASH with F2‐3 fibrosis upon demonstrating MASH resolution and fibrosis improvement [9]. Moreover, sodium‐glucose cotransporter‐2 (SGLT‐2) inhibitors also show promise: dapagliflozin achieved MASH resolution in 23% versus 8% with placebo [10] and empagliflozin reduced hepatic steatosis without affecting weight or glycemic control [11].

A recent meta‐analysis found a slightly elevated hazard ratio (HR) for major adverse liver outcomes (MALO) with SGLT‐2 inhibitors compared with GLP‐1 RAs (HR 1.07, 95% confidence interval (CI) 0.98–1.18, *I* ^2^ = 39.7%), irrespective of MASLD status [12], but was limited to only four studies restricted to US [13, 14, 15] and Korean [16] data. Understanding the comparative effectiveness between both drug classes might help to optimise treatment with respect to liver‐related outcomes, while preserving the established cardiovascular benefits [17]. Therefore, our study aimed to assess the comparative effectiveness of GLP‐1 RAs compared with SGLT‐2 inhibitors on incident MALOs, incident liver cirrhosis and all‐cause mortality among people with type 2 diabetes and MASLD/MASH.

## 2 Materials and Methods

**FIGURE 1:**
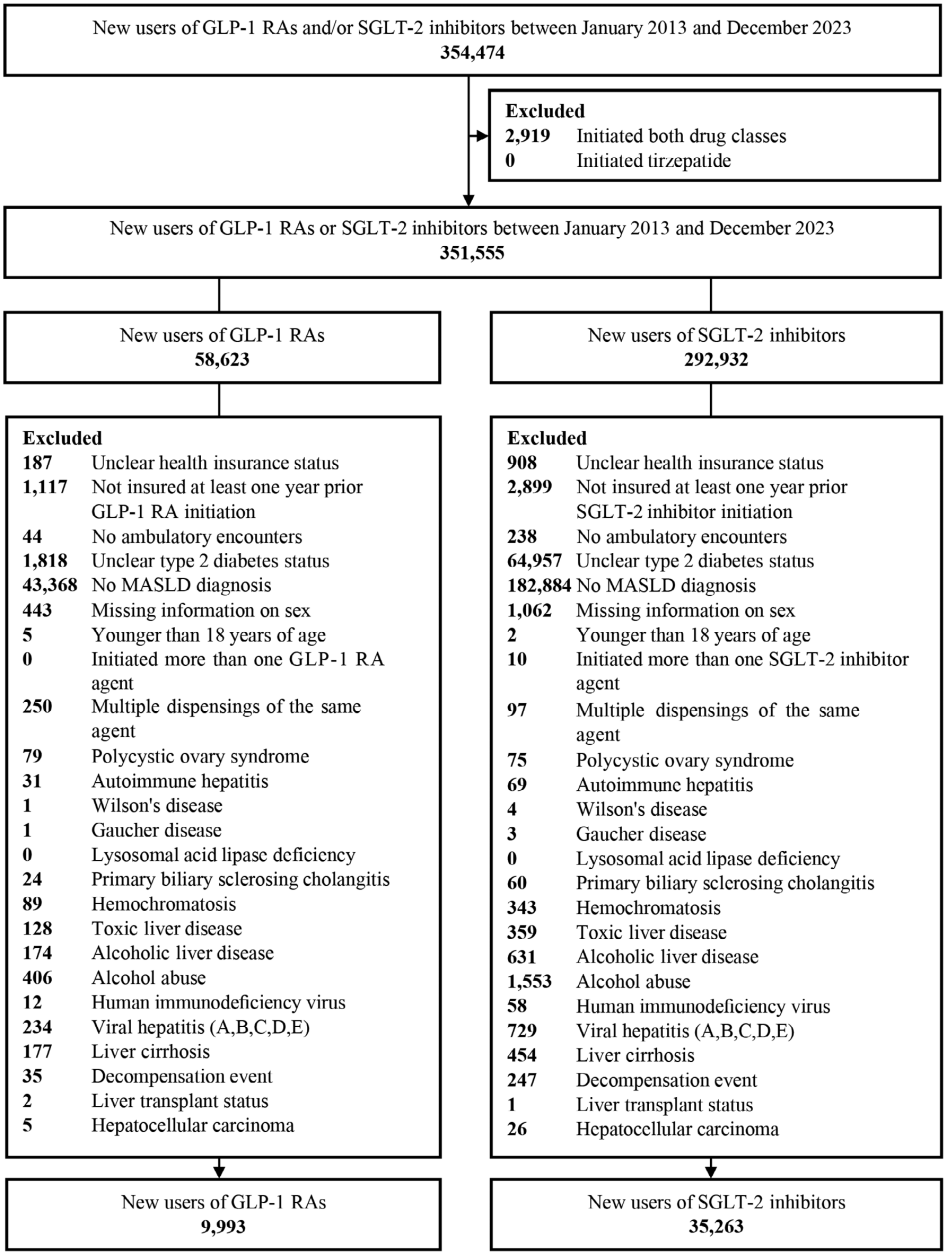
Flowchart illustrating the application of exclusion criteria and the corresponding number of people excluded at each step, resulting in the final cohort of new users of GLP‐1 RAs and SGLT‐2 inhibitors. GLP‐1 RA, glucagon‐like peptide‐1 receptor agonist; SGLT‐2, sodium‐glucose cotransporter‐2.

We conducted a retrospective cohort study and implemented an active comparator, new‐user design using nationwide health claims data. We identified people who were new users of GLP‐1 RAs (dulaglutide, semaglutide, liraglutide, exenatide, lixisenatide or albiglutide) or SGLT‐2 inhibitors (dapagliflozin, empagliflozin, ertugliflozin or canagliflozin) between January 1, 2013 and December 31, 2023 (see Figure 1 for the study selection flowchart). The cohort index date was defined as the date of the first drug dispensation that led to initiation of the respective drug. We excluded people who initiated both drug classes simultaneously, those who had initiated tirzepatide (a dual glucose‐dependent insulinotropic polypeptide (GIP) and GLP‐1 receptor agonist), had an unclear health insurance status, less than 1 year of insurance coverage prior to the cohort index date, unclear type 2 diabetes status or no MASLD/MASH diagnosis within 1 year up to the cohort index date. We defined MASLD/MASH based on ICD‐10‐GM codes (K76.0 and K75.8), consistent with previous database studies that have used ICD‐10 coding [18, 19]. Further, we excluded people with missing information on sex, those younger than 18 years of age or those who had more than one agent and/or multiple dispensings of GLP‐1 RAs or SGLT‐2 inhibitors on the cohort index date. Further, we excluded people with at least one present diagnosis of the following conditions within 1 year up to the cohort index date: polycystic ovary syndrome, autoimmune hepatitis, Wilson's disease, Gaucher disease, lysosomal acid lipase deficiency, primary biliary sclerosing cholangitis, hemochromatosis, toxic liver disease, alcoholic liver disease, alcohol abuse, human immunodeficiency virus, viral hepatitis, liver cirrhosis, decompensation event, liver transplantation or hepatocellular carcinoma (HCC). For definitions of type 2 diabetes and exclusion criteria, see Tables S1 and S2. This observational study was conducted and reported according to the STROBE guideline for observational studies [20].

### 2.1 Sources of Data

Our study utilised administrative data from BARMER, a major statutory health insurance fund in Germany. The database provides comprehensive longitudinal data collected from 2005 onwards, including detailed information on all inpatient and outpatient medical encounters, among other clinical data. Diagnoses are encoded according to the German version of the ICD (ICD‐10‐GM) and all procedures are captured by the German procedure classification ‘Operationen‐ und Prozedurenschlüssel’ (OPS). The database also contains information on dispensed medications, classified by the Anatomical Therapeutic Chemical Classification System (ATC).

### 2.2 Follow‐Up

Follow‐up started 1 day after the date of the initial drug dispensation using an intention‐to‐treat approach. Persons were followed until death, the occurrence of one of the clinical outcomes, the end of health plan enrolment or the end of study (December 31, 2024).

### 2.3 Potential Confounders

We adjusted for a wide range of potential confounders (detailed in Table S3), including sociodemographic factors (age, sex, federal state, year of cohort entry), diabetes‐related complications, cardiorenal, metabolic and other comorbidities, procedures, concomitant glucose‐lowering drugs, other drug prescriptions, drugs associated with MASLD [21], healthcare utilisation and preventive care participation. Most confounders were assessed within 1 year up to the index date, except sociodemographic characteristics evaluated at the index date and duration of MASLD, type 2 diabetes and bariatric surgery evaluated using all‐available history.

### 2.4 Weighting

We used propensity score methodology and weighted persons using matching weights [22]. In detail, persons were weighted based on the predicted probability of initiating a GLP‐1 RA rather than an SGLT‐2 inhibitor, estimated through multivariable logistic regression conditional on the covariates. Each person was weighted using the ratio of the minimum probability of treatment assignment to the actual treatment probability.

This approach estimates treatment effects in the subpopulation with greatest equipoise, focusing on individuals with propensity scores closest to 0.5 [23].

### 2.5 Primary and Secondary Outcomes

The primary outcome was a composite of MALO, consisting of hepatic decompensation events, liver transplantation and HCC. Secondary outcomes included the individual components of the MALO composite, as well as incident liver cirrhosis and all‐cause mortality. For a detailed list of outcome definitions, refer to Table S4.

### 2.6 Subgroup and Sensitivity Analyses

We conducted multiple subgroup analyses to assess possible effect measure modification by sex, cardiovascular disease (CVD), renal failure, insulin and analogues, statins, obesity (obesity was identified based on a recorded ICD‐10‐GM diagnosis (E66), as BMI values are not available in the health claims data used for this study), myocardial infarction, heart failure and kidney disease (including renal failure and nephropathy). We also assessed potential effect measure modification by the calendar era of the cohort index date (before vs. after semaglutide became available) and performed agent‐specific subgroup analyses for individual GLP‐1 RAs.

Further, we performed six sensitivity analyses. First, we censored upon tirzepatide initiation. Second, we used an on‐treatment approach with a 180‐day refill interval, censoring for drug discontinuation or switching to the comparator. Third, we extended the on‐treatment approach by additionally censoring upon tirzepatide initiation. Fourth, using standardised morbidity ratio weighting (SMRW), we targeted the average treatment effect in the treated [24]. Fifth, we used inverse probability of treatment weighting (IPTW) for the average treatment effect [23]. Sixth, we restricted outcomes to hospital diagnoses in primary position to address potential misclassification.

### 2.7 Statistical Analysis

Each baseline characteristic was summarised before and after weighting. Binary and categorical variables are presented as number and proportion (*N*, %) and continuous variables as mean and standard deviation. The balance of the characteristics between both treatment groups was assessed using absolute standardised differences (ASD) with a difference of ≥ 10% considered an imbalanced characteristic [25]. We calculated the weighted number of events per 1000 person‐years based on the Poisson distribution with respective 95% CIs. We used weighted Cox proportional hazard regression models to estimate HRs as well as their 95% CIs. Absolute risks were based on weighted Kaplan–Meier curves. Finally, risk differences (RD) were estimated at yearly intervals up to 10 years of follow‐up, with bootstrapped 95% CIs based on 1000 iterations. All statistical analyses were conducted using R (R Foundation for Statistical Computing, Vienna, Austria, http://www.r‐project.org).

## 3 Results

We identified 354 474 new users of GLP‐1 RAs and/or SGLT‐2 inhibitors between January 1, 2013 and December 31, 2023. After the exclusion criteria were applied, the cohort consisted of 9993 new users of GLP‐1 RAs and 35 263 new users of SGLT‐2 inhibitors. In the unweighted cohort, new users of GLP‐1 RAs were younger compared with new users of SGLT‐2 inhibitors (mean 61.6 vs. 67.5 years), were more likely to be female (55.2% vs. 46.4%) and had more dispensations of insulins and analogues (mean 3.0 vs. 1.5) (see Table S5). After applying matching weights, the baseline characteristics between both treatment groups were well balanced with every ASD below 10% (Table 1). Among new users of GLP‐1 RAs, the most dispensed agents were dulaglutide (44.8%), semaglutide (27.0%) and liraglutide (24.7%). Among new users of SGLT‐2 inhibitors, dapagliflozin (52.0%) and empagliflozin (46.2%) were most frequently dispensed. Figure S1 shows the distribution of the propensity scores before and after weighting for both treatment groups.

**TABLE 1:** Baseline characteristics and absolute standardised differences (%) for new users of GLP‐1 RAs and SGLT‐2 inhibitors after propensity score weighting.

Characteristic	GLP‐1 RAs	SGLT‐2 inhibitors	ASD (%)
People, *n*	9484	9416	
Age, mean (SD)	62.2 (12.0)	62.1 (12.4)	1.09
Female, *n* (%)	5155 (54.4)	5096 (54.1)	0.47
Duration of diabetes, mean (SD)	8.5 (5.3)	8.5 (5.2)	0.00
Duration of MASLD, mean (SD)	7.1 (5.5)	7.1 (5.3)	0.22
Diabetes‐related complications, *n* (%)			
Nephropathy	1702 (17.9)	1691 (18.0)	0.02
Retinopathy	953 (10.0)	943 (10.0)	0.10
Polyneuropathy	2860 (30.2)	2848 (30.2)	0.18
Foot syndrome	1909 (20.1)	1900 (20.2)	0.11
Comorbidities, *n* (%)			
Renal failure	2700 (28.5)	2644 (28.1)	0.87
Stroke	542 (5.7)	533 (5.7)	0.23
Angina pectoris	465 (4.9)	462 (4.9)	0.06
Myocardial infarction	455 (4.8)	455 (4.8)	0.17
Acute ischemic heart disease	63 (0.7)	62 (0.7)	0.06
Chronic ischemic heart disease	2284 (24.1)	2255 (23.9)	0.32
Heart failure	1764 (18.6)	1706 (18.1)	1.25
Atherosclerosis of extremities	695 (7.3)	685 (7.3)	0.21
Peripheral vascular disease	458 (4.8)	450 (4.8)	0.23
Hypertension	8488 (89.5)	8431 (89.5)	0.12
COPD	1430 (15.1)	1415 (15.0)	0.16
Osteoporosis	624 (6.6)	605 (6.4)	0.64
Dyslipidemia	6516 (68.7)	6475 (68.8)	0.14
Cancer (excluding non‐melanoma skin cancer)	1301 (13.7)	1285 (13.6)	0.21
Nicotine dependency	1596 (16.8)	1587 (16.9)	0.08
Obesity	7274 (76.7)	7218 (76.7)	0.09
Depression and anxiety‐related disorders	4785 (50.4)	4751 (50.5)	0.02
Drug abuse	140 (1.5)	134 (1.4)	0.43
Sleep apnoe	297 (3.1)	296 (3.1)	0.02
Pancreatitis	184 (1.9)	186 (2.0)	0.31
Procedures, *n* (%)			
Revascularisation	21 (0.2)	20 (0.2)	0.14
Coronary bypass/STENT	214 (2.3)	215 (2.3)	0.18
Bariatric surgery	32 (0.3)	29 (0.3)	0.47
Co‐medication, *n* (%)			
Benzodiazepines	319 (3.4)	316 (3.4)	0.04
Antidepressants	1982 (20.9)	1971 (20.9)	0.08
Opioids	1369 (14.4)	1357 (14.4)	0.07
Anticonvulsants	895 (9.4)	890 (9.5)	0.04
ACE inhibitors	3513 (37.0)	3487 (37.0)	0.02
Angiotensin II receptor blockers	4039 (42.6)	3995 (42.4)	0.32
Beta blockers	4943 (52.1)	4883 (51.9)	0.52
Loop diuretics	2349 (24.8)	2301 (24.4)	0.77
Other diuretics	1829 (19.3)	1787 (19.0)	0.79
Anti thrombotic agents	2639 (27.8)	2593 (27.5)	0.65
Calcium channel blockers	3045 (32.1)	3023 (32.1)	0.02
Corticosteroids	60 (0.6)	59 (0.6)	0.11
Bisphosphonates	93 (1.0)	91 (1.0)	0.10
Statins	4471 (47.1)	4452 (47.3)	0.28
Fibrates	178 (1.9)	183 (1.9)	0.46
Systemic corticosteroids	1098 (11.6)	1100 (11.7)	0.34
Tetracyclines	319 (3.4)	321 (3.4)	0.27
Tamoxifen	49 (0.5)	47 (0.5)	0.12
Methotrexate	135 (1.4)	137 (1.5)	0.26
Amiodaron	59 (0.6)	57 (0.6)	0.20
Obeticholic acid	0 (0.0)	0 (0.0)	0.01
Glucose‐lowering drugs, *n* (%)			
Metformin	7373 (77.7)	7356 (78.1)	0.93
Alpha glucosidase inhibitors	44 (0.5)	43 (0.5)	0.15
Sulfonylureas	865 (9.1)	862 (9.2)	0.13
DPP‐4 inhibitors	4118 (43.4)	4162 (44.2)	1.57
Insulin and analogues	3880 (40.9)	3883 (41.2)	0.65
Total dispensations of DPP‐4 inhibitors and sulfonylureas, mean (SD)	1.7 (2.2)	1.7 (2.2)	1.27
Total dispensations of insulin and analogues, mean (SD)	2.8 (4.8)	2.9 (4.9)	0.83
Health‐seeking behaviour, *n* (%)			
Influenza vaccination	3552 (37.4)	3533 (37.5)	0.14
Breast cancer screening	384 (4.0)	376 (4.0)	0.24
Neoplasm screening for men	1058 (11.2)	1064 (11.3)	0.44
Colonoscopy	61 (0.6)	61 (0.7)	0.12
Neoplasm screening for women	575 (6.1)	568 (6.0)	0.14
Skin cancer screening	951 (10.0)	943 (10.0)	0.03
Cohort entry year, *n* (%)			
2013	323 (3.4)	305 (3.2)	0.89
2014	375 (4.0)	397 (4.2)	1.29
2015	537 (5.7)	545 (5.8)	0.52
2016	630 (6.6)	634 (6.7)	0.37
2017	623 (6.6)	624 (6.6)	0.20
2018	755 (8.0)	751 (8.0)	0.05
2019	1051 (11.1)	1035 (11.0)	0.28
2020	1147 (12.1)	1148 (12.2)	0.29
2021	1483 (15.6)	1472 (15.6)	0.01
2022	1239 (13.1)	1219 (12.9)	0.35
2023	1321 (13.9)	1287 (13.7)	0.75
Hospital admissions, *n* (%)			
0	5998 (63.2)	5984 (63.5)	0.65
1	2138 (22.5)	2105 (22.4)	0.45
2	801 (8.4)	787 (8.4)	0.31
≥ 3	548 (5.8)	540 (5.7)	0.17
Outpatient admissions, *n* (%)			
≥ 1 and ≤ 10	1681 (17.7)	1666 (17.7)	0.08
≥ 11 and ≤ 20	4650 (49.0)	4619 (49.1)	0.06
≥ 21 and ≤ 30	2380 (25.1)	2366 (25.1)	0.06
> 30	773 (8.2)	765 (8.1)	0.09
Federal state, *n* (%)			
Unknown	26 (0.3)	28 (0.3)	0.39
Schleswig‐Holstein	216 (2.3)	218 (2.3)	0.25
Hamburg (Hanseatic City)	108 (1.1)	110 (1.2)	0.31
Lower Saxony	832 (8.8)	825 (8.8)	0.06
Bremen (Hanseatic City)	23 (0.2)	21 (0.2)	0.23
North Rhine‐Westphalia	2323 (24.5)	2307 (24.5)	0.03
Hesse	772 (8.1)	782 (8.3)	0.60
Rhineland‐Palatinate	416 (4.4)	414 (4.4)	0.05
Baden‐Württemberg	463 (4.9)	458 (4.9)	0.09
Bavaria (Free State)	1218 (12.8)	1201 (12.8)	0.25
Saarland	136 (1.4)	130 (1.4)	0.42
Berlin	483 (5.1)	472 (5.0)	0.37
Brandenburg	651 (6.9)	655 (7.0)	0.34
Mecklenburg‐Western Pomerania	448 (4.7)	451 (4.8)	0.31
Saxony (Free State)	508 (5.4)	495 (5.3)	0.42
Saxony‐Anhalt	487 (5.1)	481 (5.1)	0.10
Thuringia (Free State)	376 (4.0)	368 (3.9)	0.27

Abbreviations: ACE, angiotensin‐converting enzyme; ASD, absolute standardised difference; COPD, chronic obstructive pulmonary disease; DPP‐4, dipeptidyl peptidase‐4; GLP‐1 RA, glucagon‐like peptide‐1 receptor agonist; MASLD, metabolic dysfunction‐associated steatotic liver disease; SD, standard deviation; SGLT‐2, sodium‐glucose cotransporter‐2.

### 3.1 Follow‐Up Time and Censoring Reasons

For the primary composite outcome, MALO, median (interquartile range) follow‐up time was 4.3 (2.6–6.8) years. The most frequent reason for censoring was the end of the study (88.4%), followed by death (8.7%) and the end of insurance coverage (3.0%).

### 3.2 Primary Outcome

**FIGURE 2:**
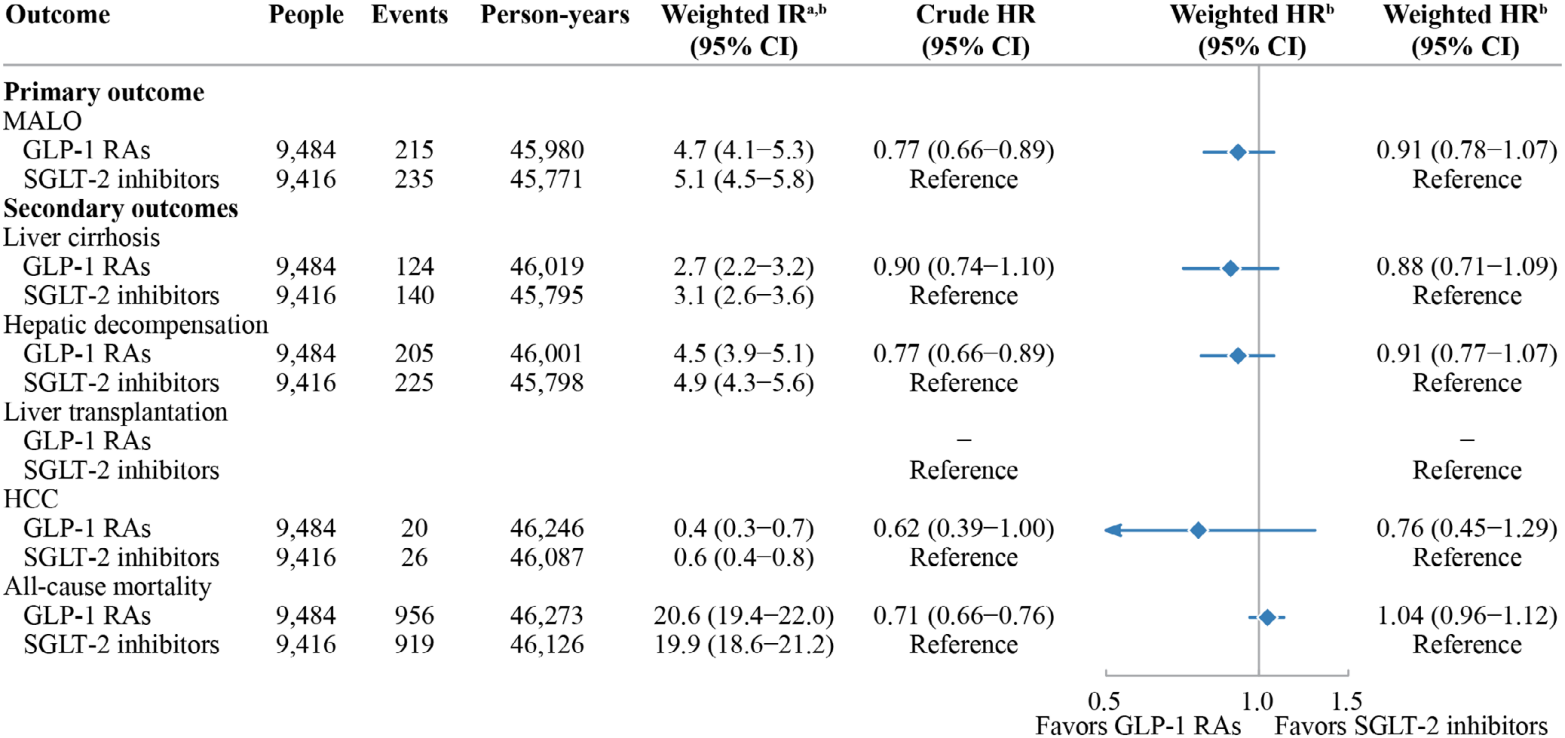
Forest plot and summary table comparing new users of GLP‐1 RAs with new users of SGLT‐2 inhibitors. The figure presents the number of participants, events, person‐years, incidence rates and hazard ratios for the primary outcome and secondary outcomes. Error bars indicate 95% confidence intervals. ^a^per 1000 person‐years. ^b^weighted using matching weights. CI, confidence interval; GLP‐1 RA, glucagon‐like peptide‐1 receptor agonist; HCC, hepatocellular carcinoma; HR, hazard ratio; IR, incidence rate; MALO, major adverse liver outcomes; SGLT‐2, sodium‐glucose cotransporter‐2.

**FIGURE 3:**
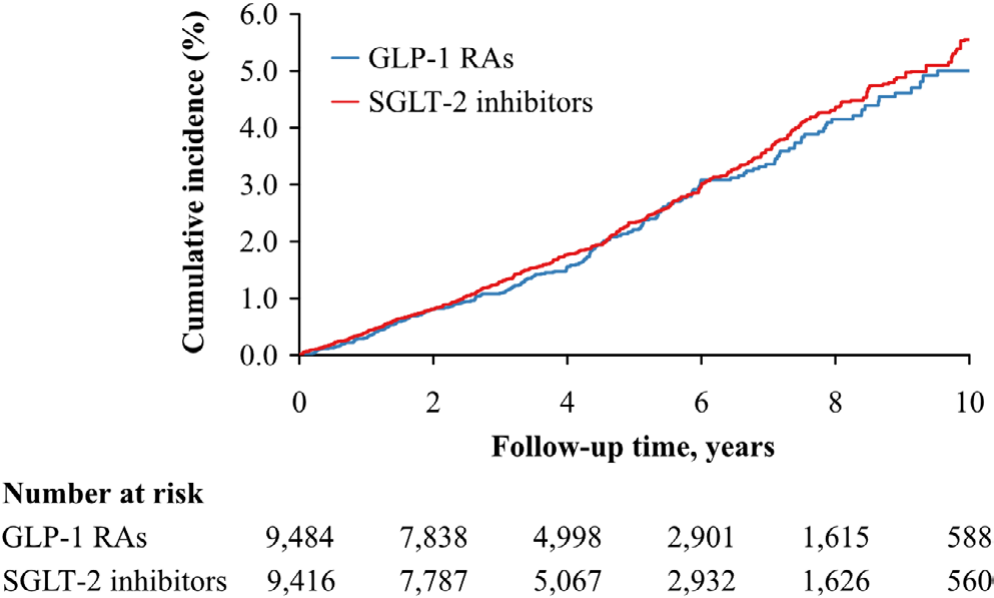
Weighted cumulative incidence curves of the primary composite outcome MALO among new users of GLP‐1 RAs and new users of SGLT‐2 inhibitors. GLP‐1 RA, glucagon‐like peptide‐1 receptor agonist; MALO, major adverse liver outcomes; SGLT‐2, sodium‐glucose cotransporter‐2.

The weighted cohort showed a lower hazard for the composite outcome MALO, with 215 and 235 events (4.7 vs. 5.1 events per 1000 person‐years) for new users of GLP‐1 RAs and SGLT‐2 inhibitors, respectively, with a weighted HR of 0.91 (95% CI 0.78–1.07). Weighted absolute risks at 1 year were 0.3% versus 0.4% for new users of GLP‐1 RA and SGLT‐2 inhibitors, respectively (RD −0.1%, 95% CI −0.2% to 0.0%). By 5 years, absolute risks increased to 2.2% versus 2.3%, respectively (RD −0.1%, 95% CI −0.6% to 0.3%). The crude and weighted HRs are shown in Figure 2. Yearly estimates of the weighted absolute risks and RDs are shown in Table S6. Details of the crude analyses are shown in Table S7. Figure 3 shows the weighted cumulative incidence curves for the primary composite outcome MALO.

### 3.3 Secondary Outcomes

For incident liver cirrhosis, new users of GLP‐1 RA had 124 events (2.7 per 1000 person‐years) while new users of SGLT‐2 inhibitors had 140 events (3.1 per 1000 person‐years; weighted HR 0.88, 95% CI 0.71–1.09). For hepatic decompensation events, new users of GLP‐1 RA had 205 events (4.5 per 1000 person‐years) compared to 225 events in new users of SGLT‐2 inhibitors (4.9 per 1000 person‐years; weighted HR 0.91, 95% CI 0.77–1.07). Liver transplantation was rare, with only 2 events observed in the unweighted SGLT‐2 inhibitor group and none in the GLP‐1 RA group. Due to the low event count, no comparative analysis could be performed. For HCC, 20 events occurred in the GLP‐1 RA group versus 26 in the SGLT‐2 inhibitor group (0.4 vs. 0.6 per 1000 person‐years; weighted HR 0.76, 95% CI 0.45–1.29). Finally, all‐cause mortality was similar between both treatment groups, with 956 events in the GLP‐1 RA group and 919 events in the SGLT‐2 inhibitor group (20.6 vs. 19.9 per 1000 person‐years, respectively; weighted HR 1.04, 95% CI 0.96–1.12). For detailed results, see Figure 2. The weighted cumulative incidence curves are shown in Figures S2–S5.

### 3.4 Subgroup and Sensitivity Analyses

Subgroup analyses revealed treatment effect modification for the primary composite outcome MALO by renal failure status at baseline (*p*‐interaction = 0.0081), with greater benefits for new users of GLP‐1 RAs observed in people without renal failure (weighted HR 0.73, 95% CI 0.59–0.91) compared to those with renal failure (weighted HR 1.14, 95% CI 0.89–1.45). Kidney disease, which additionally included nephropathy, revealed effect measure modification with weighted HRs of 1.07 (95% CI 0.85–1.35) for those with kidney disease and 0.75 (95% CI 0.59–0.94) for those without kidney disease (*p*‐interaction = 0.032). No effect measure modification was observed for sex, CVD, insulin and analogues, statins, obesity, myocardial infarction, heart failure and calendar era of the index date. Subgroup analyses by individual GLP‐1 RA agents suggested a protective association for semaglutide and liraglutide with weighted HRs of 0.74 (95% CI 0.48–1.12) and 0.74 (95% CI 0.56–0.96), respectively, whereas dulaglutide showed an increased hazard (weighted HR 1.14, 95% CI 0.93–1.39). In general, most estimates were imprecise, as indicated by the relatively wide confidence intervals. The subgroup analyses are presented in Figure S6.

In sensitivity analyses, censoring upon tirzepatide initiation yielded a weighted HR of 0.92 (95% CI 0.78–1.08). The on‐treatment exposure definition resulted in a weighted HR of 0.78 (95% CI 0.58–1.03), which remained stable at 0.78 (95% CI 0.58–1.04) when additionally censoring upon tirzepatide initiation. Reweighting the cohort using SMRW and IPTW showed similar results with weighted HRs of 0.88 (95% CI 0.73–1.05) and 0.91 (95% CI 0.75–1.10), respectively. Restricting the analysis to outcomes recorded as primary diagnoses during inpatient hospitalisations yielded a weighted HR of 0.77 (95% CI 0.56–1.07). The results of the sensitivity analyses are presented in Tables S8–S15, with Tables S10 and S12 showing the reasons for censoring for both on‐treatment exposure definitions and their relative proportions by treatment group.

## 4 Discussion

This study suggests that GLP‐1 RA initiation in people with type 2 diabetes and MASLD is associated with a potentially lower hazard compared with SGLT‐2 inhibitors for the primary composite outcome MALO. The estimated association for MALO (HR 0.91) was robust across several sensitivity analyses including censoring on tirzepatide initiation (HR 0.92) and an on‐treatment exposure definition (HR 0.78). Similar associations were observed for other individual outcomes of liver cirrhosis, hepatic decompensation events and incident HCC. For the secondary outcome all‐cause mortality, no difference was observed between the two treatment groups. Notably, the magnitude of benefits observed in crude analyses was substantially attenuated after weighting the cohort suggesting a considerable portion of the initially observed effect can be attributed to confounding. For liver transplantation, only 2 events in the unweighted SGLT‐2 inhibitor group occurred, precluding comparative analysis.

Previous studies have shown mixed results with lower hazards for GLP‐1 RAs for incident hepatic decompensation in US people with cirrhosis (HR 0.89, 95% CI 0.62–1.28) [13]. Analyses based on the TriNetX database in people with MASLD reported similar trends both for hepatic decompensation (HR 0.87, 95% CI 0.75–1.02) [14] and for incident MALO (HR 0.84, 95% CI 0.73–0.97) [15] when compared with SGLT‐2 inhibitors. The effect estimate for our primary outcome, MALO (HR 0.91), is consistent with these findings. On the other hand, in a Korean MASLD population, SGLT‐2 inhibitors were associated with a lower hazard of incident hepatic decompensation compared with GLP‐1 RAs (HR 0.93, 95% CI 0.76–1.14) [16]. The studies differed substantially among the MASLD populations analysed. Bea et al. [16] excluded individuals with alcoholic liver disease. In contrast, Wang et al. [14] did not exclude alcohol‐related conditions and derived MASLD/MASH‐specific estimates from a small subcohort (6.4% of the original cohort). Bea et al. [16] reported a lower hazard of hepatic decompensation with SGLT‐2 inhibitors compared with GLP‐1 RAs. However, semaglutide was not included due to reimbursement restrictions in Korea, which may have influenced these findings and limits generalizability to current clinical practice.

In our cohort, semaglutide accounted for more than one‐quarter of GLP‐1 RA initiations. In subgroup analyses, semaglutide was associated with a more pronounced effect compared with SGLT‐2 inhibitors (HR 0.74), which also held true for liraglutide (HR 0.74). These results are exploratory and should be interpreted cautiously, but are supported by RCT evidence for both liraglutide [26] and semaglutide [9]. Weight reduction represents a key therapeutic target in MASLD, with dose‐dependent benefits on liver histology [27]. GLP‐1 RAs such as semaglutide could be shown to achieve a mean weight loss of 11.7% in patients with MASLD/MASH, regardless of type 2 diabetes status [28]. In contrast, SGLT‐2 inhibitors have more modest effects, with a mean body weight reduction of 1.79 kg versus placebo [29], substantially lower than that observed with GLP‐1 RAs [30]. Despite this, SGLT‐2 inhibitors reduce liver lipid content and serum liver transaminases [31] and empagliflozin has been shown to reduce hepatic steatosis, even in the absence of relevant weight loss [11]. More recently, dapagliflozin was shown to achieve MASH resolution and fibrosis regression in a randomised placebo‐controlled trial [10]. While our analysis suggests a reduced hazard for incident liver cirrhosis with GLP‐1 RAs (HR 0.88), semaglutide did not improve fibrosis or resolve MASH in individuals with established cirrhosis despite shown to be safe [32]. Tirzepatide showed efficacy for MASH resolution and fibrosis reduction versus placebo in a phase 2b RCT [33]. Subsequent tirzepatide initiation occurred in 8.3% and 1.6% of our cohort in intention‐to‐treat and on‐treatment analyses, respectively, but sensitivity analyses censoring at initiation yielded identical results, indicating no confounding effect.

### 4.1 Strengths and Limitations

This study has specific strengths. First, we implemented an active comparator, new‐user design and utilised a large German cohort based on routine care claims, which provides high external validity and reflects real‐world clinical practice. Second, we anticipated people to initiate tirzepatide during their follow‐up and included sensitivity analyses where we additionally censored upon tirzepatide initiation in the intention‐to‐treat as well as the on‐treatment exposure definitions. Third, unlike Kuo et al. [15], who excluded people at baseline who switched or augmented treatments during follow‐up which may have introduced bias, we conducted an intention‐to‐treat analysis and a sensitivity analysis using an on‐treatment exposure definition to address potential exposure misclassification, which strengthened the robustness of our findings.

Our study also has limitations. First, defining outcomes using claims data presents inherent challenges and no external validation studies exist for our dataset. We defined outcomes based on hospital diagnoses (ICD‐10‐GM coded) and further minimised potential misclassification by considering only diagnoses recorded in the primary position in a sensitivity analysis. Second, the validity of ICD‐10‐GM codes to identify MASLD/MASH has not been externally validated in our dataset. Documented cases would suggest a low prevalence in Germany [34], which contrasts with the high prevalence estimated in people with type 2 diabetes [1]. Thus, our cohort may represent a selective sample in which MASLD/MASH diagnoses may have been driven by certain clinical symptoms, diagnostic procedures with accidental liver findings and/or by physicians with particularly thorough documentation practices. Third, as our study used observational data, we cannot exclude residual confounding, even after careful adjustment for multiple covariates. Indeed, the effect estimates were larger in unadjusted analyses and attenuated after adjustment, suggesting that confounding explained much of the observed effect. Consequently, it is possible that unmeasured confounders could further attenuate the effect in the absence of data on lifestyle and socioeconomic status.

In subgroup analyses, the association appeared more pronounced in people without renal impairment than in those with renal impairment. Although people with renal impairment represent a clinically important subgroup, this finding should be interpreted cautiously, as the estimate in this subgroup was less precise and does not necessarily indicate a clinically meaningful difference in treatment effectiveness. From a pharmacological perspective, the difference is unlikely to be explained by altered pharmacokinetics, as most long‐acting human GLP‐1 RAs, including liraglutide and semaglutide, do not show clinically meaningful changes in exposure across stages of chronic kidney disease and generally do not require dose adjustment [35, 36]. Available evidence also suggests that pharmacodynamic effects are largely preserved in chronic kidney disease, with semaglutide maintaining glucose‐lowering efficacy across eGFR strata [37]. Current pharmacokinetic and pharmacodynamic evidence therefore does not support a biologically plausible kidney‐specific loss of GLP‐1 RA activity. A more plausible explanation is confounding by indication. In clinical practice, people with more advanced renal impairment may be preferentially channelled toward GLP‐1 RAs rather than SGLT‐2 inhibitors, as the glycaemic efficacy of SGLT‐2 inhibitors declines with worsening kidney function [38]. In line with this, current ADA guidance recommends GLP‐1 RAs as the preferred glucose‐lowering therapy in adults with type 2 diabetes and advanced chronic kidney disease [39]. Because renal impairment was defined using ICD‐10‐GM codes in our study rather than measured eGFR, we were unable to account for the severity of kidney dysfunction and initiators of GLP‐1 RAs in this subgroup may therefore have had more severe underlying renal impairment compared with initiators of SGLT‐2 inhibitors. Such imbalance could attenuate an apparent protective association of GLP‐1 RAs in this subgroup. Fourth, although we excluded people with alcoholic liver disease and alcohol abuse to better approximate a MASLD population, routine healthcare data lack detailed information on quantitative alcohol consumption so that people with metabolic and alcohol‐associated liver disease (MetALD) could not be completely excluded in our cohort. Restricting to ICD‐10‐GM codes may only have excluded cases severe enough to prompt physician documentation. Fifth, we were not able to investigate possible effect measure modification by indication because GLP‐1 RAs prescribed solely for weight loss are not reimbursed by the German statutory health insurance and their indication is not reliably recorded in the claims data. This could be relevant, as treatment effects may differ between doses used for obesity versus type 2 diabetes. Finally, we lacked data to calculate fibrosis risk scores, such as FIB‐4, limiting our ability to assess whether baseline fibrosis severity was comparable between both treatment groups. Notably, prior studies have shown that higher fibrosis risk scores are associated with increased all‐cause mortality in persons with type 2 diabetes [40]. Mortality rates in MASLD rise with fibrosis stage, ranging from 0.32, 0.89 and 1.76 deaths per 100 person‐years for F0–F2, F3 and F4, respectively [41]. In our cohort, we observed no difference in all‐cause mortality between GLP‐1 RAs and SGLT‐2 inhibitors (HR 1.04), consistent with Krishnan et al. [19] who reported an HR of 1.06 (95% CI 0.97–1.15) in a similar MASLD population. These findings may suggest that differences in baseline fibrosis stage are unlikely to fully explain our results, although this remains speculative in the absence of direct fibrosis staging data.

## 5 Conclusion

In conclusion, our results suggest a potentially lower hazard of MALO with GLP‐1 RAs compared with SGLT‐2 inhibitors, consistent across various sensitivity analyses, though estimates were imprecise with wide confidence intervals. As this study used an active comparator design, these findings reflect differences between two active treatment strategies and do not establish the effectiveness of GLP‐1 RAs on liver outcomes compared with no treatment. Further research may also focus on selective active ingredients of GLP‐1 RAs and additionally investigate the effectiveness of tirzepatide in routine care.

## Author Contributions

G.A.M., R.A.H., M.R., W.R. and O.K. were involved in the conception, design and conduct of the study and the analysis and interpretation of the results. G.A.M. and R.A.H. did the statistical analysis. G.A.M. wrote the first draft of the manuscript and all authors edited, reviewed and approved the final version of the manuscript.

G.A.M. and O.K. are the guarantors of this work and, as such, had full access to all the data in the study and take responsibility for the integrity of the data and the accuracy of the data analysis.

## Funding

The authors have nothing to report.

## Conflicts of Interest

M.R. received personal fees for lectures or advisory boards from Astra Zeneca, Boehringer‐Ingelheim, Echosens, Eli Lilly, Madrigal, MSD, Novo Nordisk and Target RWE and investigator‐initiated research support from Boehringer‐Ingelheim and Novo Nordisk to the German Diabetes Center (DDZ). W.R. reports the receipt of consulting fees for attending educational sessions by Novo Nordisk outside of the topic of the current work. O.K. received honoraria for biostatistical education from Berlin‐Chemie. G.A.M. and R.A.H. have no conflicts of interest.

## Supporting information


**Table S1:** Definition of type 2 diabetes.
**Table S2:** Exclusion criteria.
**Table S3:** Definition of covariates.
**Table S4:** Definition of study outcomes.
**Table S5:** Baseline characteristics and corresponding absolute standardised differences (%) for new users of GLP‐1 RAs and SGLT‐2 inhibitors before propensity score weighting.
**Table S6:** Weighted absolute risks and risk differences for the primary outcome MALO comparing new users of GLP‐1 RAs with SGLT‐2 inhibitors after propensity score weighting.
**Table S7:** Results for the primary outcome and secondary outcomes comparing new users of GLP‐1 RAs with SGLT‐2 inhibitors before propensity score weighting (Intention‐to‐treat exposure definition).
**Table S8:** Results for the primary outcome and secondary outcomes comparing new users of GLP‐1 RAs with SGLT‐2 inhibitors after propensity score weighting (Intention‐to‐treat exposure definition, censoring upon tirzepatide initiation).
**Table S9:** Results for the primary outcome and secondary outcomes comparing new users of GLP‐1 RAs with SGLT‐2 inhibitors after propensity score weighting (on‐treatment exposure definition).
**Table S10:** Censoring reasons and their relative proportions for the on‐treatment exposure definition for both treatment groups of new users of GLP‐1 RAs and SGLT‐2 inhibitors.
**Table S11:** Results for the primary outcome and secondary outcomes comparing new users of GLP‐1 RAs with SGLT‐2 inhibitors after propensity score weighting (on‐treatment exposure definition, censoring upon tirzepatide initiation).
**Table S12:** Censoring reasons and their relative proportions for the on‐treatment exposure definition (including censoring upon tirzepatide initiation) for both treatment groups of new users of GLP‐1 RAs and SGLT‐2 inhibitors.
**Table S13:** Results for the primary outcome and secondary outcomes comparing new users of GLP‐1 RAs with SGLT‐2 inhibitors after propensity score weighting (Standardised morbidity ratio weighting).
**Table S14:** Results for the primary outcome and secondary outcomes comparing new users of GLP‐1 RAs with SGLT‐2 inhibitors after propensity score weighting (Inverse probability of treatment weighting).
**Table S15:** Results for the primary outcome and secondary outcomes comparing new users of GLP‐1 RAs with SGLT‐2 inhibitors after propensity score weighting (Restricting outcomes to inpatient diagnoses in primary position).
**Figure S1:** Propensity score distribution before weighting on the propensity score (A) and after weighting using matching weights (B) for new users of GLP‐1 RAs and SGLT‐2 inhibitors.
**Figure S2:** Weighted cumulative incidence curves of incident liver cirrhosis among new users of GLP‐1 RAs and SGLT‐2 inhibitors.
**Figure S3:** Weighted cumulative incidence curves of incident hepatic decompensation among new users of GLP‐1 RAs and SGLT‐2 inhibitors.
**Figure S4:** Weighted cumulative incidence curves of hepatocellular carcinoma among new users of GLP‐1 RAs and SGLT‐2 inhibitors.
**Figure S5:** Weighted cumulative incidence curves of all‐cause mortality among new users of GLP‐1 RAs and SGLT‐2 inhibitors.
**Figure S6:** Primary analysis and each subgroup analysis for new users of GLP‐1 RAs compared with SGLT‐2 inhibitors for the primary composite outcome major adverse liver outcomes.

## Data Availability

The study was approved by the Ethics Committee of the Medical Faculty of Heinrich Heine University Düsseldorf, Germany (approval number: 2025–3379). The datasets analysed are not publicly available, as they are the property of BARMER (Wuppertal, Germany).
