# Supplementary material for: Glucagon‐Like Peptide‐1 Receptor Agonists and Incident Major Adverse Liver Outcomes in People With Type 2 Diabetes and Metabolic Dysfunction‐Associated Steatotic Liver Disease
Source: Diabetes Obes Metab. 2026 Jun 22;28(9):8277–87. doi: 10.1111/dom.71023 (PMC13449085; doi:10.1111/dom.71023)
Supplement: Supplementary file 1 [file DOM-28-8277-s001.docx]

ONLINE-ONLY SUPPLEMENTAL MATERIAL

Glucagon-Like Peptide-1 Receptor Agonists and Incident Major Adverse Liver Outcomes in People With Type 2 Diabetes and Metabolic Dysfunction-Associated Steatotic Liver Disease

Gregor A. Maier^1^, Ramon A. Hofmann^1^, Michael Roden^2,3,4^, Wolfgang Rathmann^1^, Oliver Kuss^1,2,5^

Affiliations

^1^ Institute for Biometrics and Epidemiology, German Diabetes Center, Leibniz Center for Diabetes Research at Heinrich Heine University, Düsseldorf, Germany

^2^ German Center for Diabetes Research, Partner Düsseldorf, München-Neuherberg, Germany

^3^ Division of Endocrinology and Diabetology, Medical Faculty and University Hospital, Heinrich Heine University, University Hospital, Düsseldorf, Germany

^4^ Institute for Clinical Diabetology, German Diabetes Center, Leibniz Center for Diabetes Research at Heinrich Heine University, Düsseldorf, Germany

^5^ Centre for Health and Society, Medical Faculty and University Hospital Düsseldorf, Heinrich Heine University, Düsseldorf, Germany

Corresponding author

Gregor A. Maier, MSc

German Diabetes Center,

Institute for Biometrics and Epidemiology,

Auf’m Hennekamp 65,

40225 Düsseldorf, Germany

E-Mail: [gregor.maier@ddz.de](mailto:gregor.maier@ddz.de),

Phone: +49-(0)-211-33-82-338

ORCID

Gregor A. Maier: 0009-0000-4528-9475

Michael Roden: 0000-0001-8200-6382

Wolfgang Rathmann: 0000-0001-7804-1740

Oliver Kuß: 0000-0003-3301-5869

CONTENT

[Supplementary Tables	4](#_Toc232680266)

[Table S1. Definition of type 2 diabetes	4](#_Toc232680267)

[Table S2. Exclusion criteria	4](#_Toc232680268)

[Table S3. Definition of covariates	5](#_Toc232680269)

[Table S4. Definition of study outcomes	10](#_Toc232680270)

[Table S5. Baseline characteristics and corresponding absolute standardised differences (%) for new users of GLP-1 RAs and SGLT-2 inhibitors before propensity score weighting	10](#_Toc232680271)

[Table S6. Weighted absolute risks and risk differences for the primary outcome MALO comparing new users of GLP-1 RAs with SGLT-2 inhibitors after propensity score weighting	13](#_Toc232680272)

[Table S7. Results for the primary outcome and secondary outcomes comparing new users of GLP-1 RAs with SGLT-2 inhibitors before propensity score weighting (Intention-to-treat exposure definition)	14](#_Toc232680273)

[Table S8. Results for the primary outcome and secondary outcomes comparing new users of GLP-1 RAs with SGLT-2 inhibitors after propensity score weighting (Intention-to-treat exposure definition, censoring upon tirzepatide initiation)	15](#_Toc232680274)

[Table S9. Results for the primary outcome and secondary outcomes comparing new users of GLP-1 RAs with SGLT-2 inhibitors after propensity score weighting (on-treatment exposure definition)	16](#_Toc232680275)

[Table S10. Censoring reasons and their relative proportions for the on-treatment exposure definition for both treatment groups of new users of GLP-1 RAs and SGLT-2 inhibitors	16](#_Toc232680276)

[Table S11. Results for the primary outcome and secondary outcomes comparing new users of GLP-1 RAs with SGLT-2 inhibitors after propensity score weighting (on-treatment exposure definition, censoring upon tirzepatide initiation)	17](#_Toc232680277)

[Table S12. Censoring reasons and their relative proportions for the on-treatment exposure definition (including censoring upon tirzepatide initiation) for both treatment groups of new users of GLP-1 RAs and SGLT-2 inhibitors	17](#_Toc232680278)

[Table S13. Results for the primary outcome and secondary outcomes comparing new users of GLP-1 RAs with SGLT-2 inhibitors after propensity score weighting (Standardised morbidity ratio weighting)	18](#_Toc232680279)

[Table S14. Results for the primary outcome and secondary outcomes comparing new users of GLP-1 RAs with SGLT-2 inhibitors after propensity score weighting (Inverse probability of treatment weighting)	19](#_Toc232680280)

[Table S15. Results for the primary outcome and secondary outcomes comparing new users of GLP-1 RAs with SGLT-2 inhibitors after propensity score weighting (Restricting outcomes to inpatient diagnoses in primary position)	20](#_Toc232680281)

[Supplementary Figures	21](#_Toc232680282)

[Figure S1. Propensity score distribution before weighting on the propensity score (A) and after weighting using matching weights (B) for new users of GLP-1 RAs and SGLT-2 inhibitors	21](#_Toc232680283)

[Figure S2. Weighted cumulative incidence curves of incident liver cirrhosis among new users of GLP-1 RAs and SGLT-2 inhibitors	21](#_Toc232680284)

[Figure S3. Weighted cumulative incidence curves of incident hepatic decompensation among new users of GLP-1 RAs and SGLT-2 inhibitors	22](#_Toc232680285)

[Figure S4. Weighted cumulative incidence curves of incident hepatocellular carcinoma among new users of GLP-1 RAs and SGLT-2 inhibitors	22](#_Toc232680286)

[Figure S5. Weighted cumulative incidence curves of all-cause mortality among new users of GLP-1 RAs and SGLT-2 inhibitors	23](#_Toc232680287)

[Figure S6. Primary analysis and each subgroup analysis for new users of GLP-1 RAs compared with SGLT-2 inhibitors for the primary composite outcome major adverse liver outcomes	24](#_Toc232680288)

# Supplementary Tables

## Table S1. Definition of type 2 diabetes

|  | **E10x** | **E11x/E12x** | **E13x** | **E14x** | **Insulin and analogues** | **Glucose-lowering agents, except insulin and analogues** |
| --- | --- | --- | --- | --- | --- | --- |
| Combination 1 | No | Yes | - | - | - | - |
| Combination 2 | Yes | Yes | - | - | - | Yes |
| Combination 3 | Yes | Yes | - | - | No | - |
| Combination 4 | No | No | No | Yes | - | Yes |

'Yes' indicates the presence of a diagnosis or dispensing, 'No' indicates its absence, and '-' indicates that either presence or absence is acceptable. Outpatient diagnoses were included when coded as 'G' or 'Z', while inpatient diagnoses were considered as main diagnoses only. Individuals were excluded from the cohort if any of the defined combinations were not present prior to the cohort index date.
Table adapted from Reitzle et al. ^1^

## Table S2. Exclusion criteria

| **Criterion** | **Code(s)** | **Evaluation window** | **Evaluated in** |
| --- | --- | --- | --- |
| Combination of GLP-1 RAs and SGLT-2 inhibitors | Simultaneous initiation of GLP-1 RAs and SGLT-2 inhibitors | 0;0 | Drug dispensation dates |
| Tirzepatide | Dispensing of tirzepatide | -365;0 | Drug dispensation dates |
| Age | Under 18 years of age | 0;0 | … |
| Sex | Sex missing | 0;0 | … |
| Polycystic ovary syndrome | E282x | -365;0 | Outpatient: G, Z  Inpatient: Main- and secondary diagnoses |
| Autoimmune hepatitis | K754x | -365;0 | Outpatient: G, Z  Inpatient: Main- and secondary diagnoses |
| Wilson‘s disease | E830x | -365;0 | Outpatient: G, Z  Inpatient: Main- and secondary diagnoses |
| Gaucher disease | E752x | -365;0 | Outpatient: G, Z  Inpatient: Main- and secondary diagnoses |
| Lysosomal acid lipase deficiency | K755x | -365;0 | Outpatient: G, Z  Inpatient: Main- and secondary diagnoses |
| Primary biliary sclerosing cholangitis | K743x | -365;0 | Outpatient: G, Z  Inpatient: Main- and secondary diagnoses |
| Hemochromatosis | E831x | -365;0 | Outpatient: G, Z  Inpatient: Main- and secondary diagnoses |
| Toxis liver disease | K71x | -365;0 | Outpatient: G, Z  Inpatient: Main- and secondary diagnoses |
| Alcoholic liver disease | K70x | -365;0 | Outpatient: G, Z  Inpatient: Main- and secondary diagnoses |
| Alcohol abuse | F10x | -365;0 | Outpatient: G, Z  Inpatient: Main- and secondary diagnoses |
| Human immunodeficiency virus | B20x-B24x | -365;0 | Outpatient: G, Z  Inpatient: Main- and secondary diagnoses |
| Viral hepatitis | B15x-B19x | -365;0 | Outpatient: G, Z  Inpatient: Main- and secondary diagnoses |
| Liver cirrhosis | See outcome definition | -365;0 | Outpatient: G, Z  Inpatient: Main- and secondary diagnoses |
| Hepatic decompensation event | See outcome definition | -365;0 | Outpatient: G, Z  Inpatient: Main- and secondary diagnoses |
| Liver transplantation | See outcome definition | -365;0 | Outpatient: G, Z  Inpatient: Main- and secondary diagnoses |
| Hepatocellular carcinoma | See outcome definition | -365;0 | Outpatient: G, Z  Inpatient: Main- and secondary diagnoses |

Abbreviations: GLP-1 RA, glucagon-like peptide-1 receptor agonist; SGLT-2, sodium-glucose cotransporter-2.

## Table S3. Definition of covariates

| **Covariate** | **Code(s)** | **Evaluation window** | **Evaluated in** |
| --- | --- | --- | --- |
| Age | Age at the time of cohort index | 0;0 | … |
| Sex | Sex at the time of cohort index | 0;0 | … |
| Duration of MASLD | In years, since first diagnosis | -∞;0 | … |
| Duration of diabetes | In years, since first diagnosis | -∞;0 | … |
| Diabetes-related complications | … | … | … |
| Nephropathy ^2^ | E10-14x + .20, .21, .72, .73, .74, .75, N083x | -365;0 | Outpatient: G, Z  Inpatient: Main- and secondary diagnoses |
| Retinopathy ^3^ | H360 OR (E1x3 AND (H350 OR H352 OR H358)) | -365;0 | Outpatient: G, Z  Inpatient: Main- and secondary diagnoses |
| Polyneuropathy ^3^ | G632 OR ((E1x4 OR E1x74 OR E1x75) AND (G629 OR G633)) | -365;0 | Outpatient: G, Z  Inpatient: Main- and secondary diagnoses |
| Foot syndrome ^3^ | E1x74 OR E1x75 OR M1427 OR M1467 | -365;0 | Outpatient: G, Z  Inpatient: Main- and secondary diagnoses |
| Comorbidities | … | … | … |
| Renal failure | N17x-N19x | -365;0 | Outpatient: G, Z  Inpatient: Main- and secondary diagnoses |
| Stroke | I60x-I64x | -365;0 | Outpatient: G, Z  Inpatient: Main- and secondary diagnoses |
| Angina pectoris | I20x | -365;0 | Outpatient: G, Z  Inpatient: Main- and secondary diagnoses |
| Myocardial infarction | I21x,I22x | -365;0 | Outpatient: G, Z  Inpatient: Main- and secondary diagnoses |
| Acute ischemic heart disease | I24x | -365;0 | Outpatient: G, Z  Inpatient: Main- and secondary diagnoses |
| Chronic ischemic heart disease | I25x | -365;0 | Outpatient: G, Z  Inpatient: Main- and secondary diagnoses |
| Heart failure | I50x, I130, I110, I132 | -365;0 | Outpatient: G, Z  Inpatient: Main- and secondary diagnoses |
| Atherosclerosis of extremities | I702 | -365;0 | Outpatient: G, Z  Inpatient: Main- and secondary diagnoses |
| Peripheral vascular disease | I739 | -365;0 | Outpatient: G, Z  Inpatient: Main- and secondary diagnoses |
| Hypertension | I10x- I13x, I15x | -365;0 | Outpatient: G, Z  Inpatient: Main- and secondary diagnoses |
| COPD | J44x | -365;0 | Outpatient: G, Z  Inpatient: Main- and secondary diagnoses |
| Osteoporosis | M80x, M81x | -365;0 | Outpatient: G, Z  Inpatient: Main- and secondary diagnoses |
| Dyslipidemia | E78x | -365;0 | Outpatient: G, Z  Inpatient: Main- and secondary diagnoses |
| Cancer (excluding non-melanoma skin cancer) | C00x-C97x, except C44x | -365;0 | Outpatient: G, Z  Inpatient: Main- and secondary diagnoses |
| Nicotine dependency | F17x | -365;0 | Outpatient: G, Z  Inpatient: Main- and secondary diagnoses |
| Obesity | E66x | -365;0 | Outpatient: G, Z  Inpatient: Main- and secondary diagnoses |
| Depression and/ or anxiety-related disorders | F32x, F33x, F34x, F38x-F45x, F48x | -365;0 | Outpatient: G, Z  Inpatient: Main- and secondary diagnoses |
| Drug abuse | F11x-F16x, F18x, F19x, R78x, T40x | -365;0 | Outpatient: G, Z  Inpatient: Main- and secondary diagnoses |
| Sleep apnoe | G473x | -365;0 | Outpatient: G, Z  Inpatient: Main- and secondary diagnoses |
| Pancreatitis | K85x, K861 | -365;0 | Outpatient: G, Z  Inpatient: Main- and secondary diagnoses |
| Procedures | … | … | … |
| Revascularization | 5-361x, 5-362x,5-363x | -365;0 | OPS |
| Coronary bypass / STENT | 8-836x, 8-837x, 8-84x | -365;0 | OPS |
| Bariatric surgery | 5-434.3, 5-434.4, 5-434.5, 5-445.0, 5-445.1, 5-445.2, 5-445.4, 5-445.5, 5-448.a, 5-448.b, 5-448.c | -∞;0 | OPS |
| Co-medications | … | … | … |
| Benzodiazepine | N05BAx | -365;0 | Drug dispensation dates |
| Antidepressants | N06Ax | -365;0 | Drug dispensation dates |
| Opioids | N02Ax | -365;0 | Drug dispensation dates |
| Anticonvulsants | N03x | -365;0 | Drug dispensation dates |
| ACE inhibitors | C09Ax, C09Bx | -365;0 | Drug dispensation dates |
| Angiotensin II receptor blockers | C09Cx, C09Dx | -365;0 | Drug dispensation dates |
| ß blockers | C07x | -365;0 | Drug dispensation dates |
| Loop diuretics | C03Cx | -365;0 | Drug dispensation dates |
| Other diuretics | C03Ax, C03Bx, C03Dx | -365;0 | Drug dispensation dates |
| Anti thrombotic agents | B01x | -365;0 | Drug dispensation dates |
| Calcium channel blockers | C08x | -365;0 | Drug dispensation dates |
| Corticosteroids | A01ACx | -365;0 | Drug dispensation dates |
| Bisphosphonate | M05BAx, M05BBx | -365;0 | Drug dispensation dates |
| Statins | C10AAx | -365;0 | Drug dispensation dates |
| Fibrates | C10ABx | -365;0 | Drug dispensation dates |
| Systemic corticosteroids | H02x | -365;0 | Drug dispensation dates |
| Tetracyclines | J01Ax | -365;0 | Drug dispensation dates |
| Tamoxifen | L02BA01 | -365;0 | Drug dispensation dates |
| Methotrexate | L01BA01, L04AX03 | -365;0 | Drug dispensation dates |
| Amiodaron | C01BD01 | -365;0 | Drug dispensation dates |
| Obeticholic acid | A05AA04 | -365;0 | Drug dispensation dates |
| Glucose-lowering drugs |  |  |  |
| Metformin | Dispensation dates | -365;0 | Drug dispensation dates |
| Alpha glucosidase inhibitors | Dispensation dates | -365;0 | Drug dispensation dates |
| Sulfonylureas | Dispensation dates | -365;0 | Drug dispensation dates |
| DPP-4 inhibitors | Dispensation dates | -365;0 | Drug dispensation dates |
| Insulin and analogs | Dispensation dates | -365;0 | Drug dispensation dates |
| Total dispensations of DPP-4 inhibitors and sulfonylureas | Total dispensation dates | -365;0 | Drug dispensation dates |
| Total dispensations of insulin and analogs | Total dispensation dates | -365;0 | Drug dispensation dates |
| Health-seeking behaviour | … | … | … |
| Influenza vaccination | 89111, 89112, 89112Y | -365;0 | GOP |
| Breast cancer screening | 01750 | -365;0 | GOP |
| Neoplasm screening for men | 01731 | -365;0 | GOP |
| Colonoscopy | 01741 | -365;0 | GOP |
| Neoplasm screening for women | 01760 | -365;0 | GOP |
| Skin cancer screening | 01745, 01746 | -365;0 | GOP |
| Cohort entry year | Year of cohort entry |  |  |
| Hospital admissions | Categorized in: Zero, one, two, three or more hospital admissions one year prior cohort index | -365;0 | Inpatient |
| Outpatient admissions | Categorized in: One to ten, 11–20, 21–30, more than 30 | -365;0 | Outpatient |
| Federal state | Residency at cohort entry | 0;0 | … |

Abbreviations: MASLD, metabolic dysfunction-associated steatotic liver disease; COPD, chronic obstructive pulmonary disease; OPS, Operationen- und Prozedurenschlüssel; ACE, angiotensin-converting enzyme; DPP-4, dipeptidyl peptidase 4; GOP, Gebührenordnungsposition.

## Table S4. Definition of study outcomes

| **Outcome** | **ICD-10-GM/ OPS codes** |
| --- | --- |
| MALO | I850x, I864x, I983x, K767x, I982x, Z944, C220, C229, OPS: 5-504 |
| Liver cirrhosis | K740x, K741x, K742x, K746x, K747x |
| Decompensation events | I850x, I864x, I983x, K767x, I982x |
| Liver transplantation | Z944, OPS: 5-504x |
| HCC | C220, C229 |
| All-cause mortality | Date of death |

Abbreviations: ICD-10-GM, International Classification of Diseases, tenth revision, German modification; HCC, hepatocellular carcinoma; MALO, major adverse liver outcomes; OPS, Operationen- und Prozedurenschlüssel.

## Table S5. Baseline characteristics and corresponding absolute standardised differences (%) for new users of GLP-1 RAs and SGLT-2 inhibitors before propensity score weighting

| **Characteristic** | **GLP-1 RAs** | **SGLT-2 inhibitors** | **ASD (%)** |
| --- | --- | --- | --- |
| People, n | 9,993 | 35,263 |  |
|  |  |  |  |
| Age, mean (SD) | 61.6 (12.3) | 67.5 (11.7) | 49.23 |
| Female, n (%) | 5,513 (55.2) | 16,365 (46.4) | 17.59 |
| Duration of diabetes, mean (SD) | 8.4 (5.3) | 9.4 (5.3) | 19.33 |
| Duration of MASLD, mean (SD) | 7.0 (5.4) | 8.1 (5.6) | 19.71 |
| Diabetes-related complications, n (%) |  |  |  |
| Nephropathy | 1,826 (18.3) | 5,416 (15.4) | 7.80 |
| Retinopathy | 1,023 (10.2) | 2,856 (8.1) | 7.41 |
| Polyneuropathy | 3,067 (30.7) | 8,926 (25.3) | 12.00 |
| Foot syndrome | 2,051 (20.5) | 5,690 (16.1) | 11.36 |
| Comorbidities, n (%) |  |  |  |
| Renal failure | 2,857 (28.6) | 10,455 (29.6) | 2.33 |
| Stroke | 562 (5.6) | 2,437 (6.9) | 5.31 |
| Angina pectoris | 474 (4.7) | 2,431 (6.9) | 9.20 |
| Myocardial infarction | 460 (4.6) | 2,802 (7.9) | 13.82 |
| Acute ischemic heart disease | 64 (0.6) | 341 (1.0) | 3.66 |
| Chronic ischemic heart disease | 2,336 (23.4) | 11,785 (33.4) | 22.41 |
| Heart failure | 1,813 (18.1) | 9,377 (26.6) | 20.38 |
| Atherosclerosis of extremities | 719 (7.2) | 3,065 (8.7) | 5.54 |
| Peripheral vascular disease | 474 (4.7) | 2,016 (5.7) | 4.37 |
| Hypertension | 8,924 (89.3) | 31,926 (90.5) | 4.10 |
| COPD | 1,508 (15.1) | 5,220 (14.8) | 0.81 |
| Osteoporosis | 651 (6.5) | 2,806 (8.0) | 5.57 |
| Dyslipidemia | 6,838 (68.4) | 25,151 (71.3) | 6.32 |
| Cancer (excluding non-melanoma skin cancer) | 1,353 (13.5) | 5,813 (16.5) | 8.25 |
| Nicotine dependency | 1,684 (16.9) | 5,461 (15.5) | 3.71 |
| Obesity | 7,772 (77.8) | 19,632 (55.7) | 48.25 |
| Depression and anxiety-related disorders | 5,119 (51.2) | 15,400 (43.7) | 15.17 |
| Drug abuse | 158 (1.6) | 380 (1.1) | 4.40 |
| Sleep apnoe | 318 (3.2) | 778 (2.2) | 6.03 |
| Pancreatitis | 188 (1.9) | 877 (2.5) | 4.14 |
| Procedures, n (%) |  |  |  |
| Revascularization | 21 (0.2) | 248 (0.7) | 7.32 |
| Coronary bypass / STENT | 215 (2.2) | 1,834 (5.2) | 16.26 |
| Bariatric surgery | 46 (0.5) | 46 (0.1) | 6.08 |
| Co-medication, n (%) |  |  |  |
| Benzodiazepines | 351 (3.5) | 997 (2.8) | 3.91 |
| Antidepressants | 2,152 (21.5) | 5,787 (16.4) | 13.10 |
| Opioids | 1,477 (14.8) | 4,419 (12.5) | 6.55 |
| Anticonvulsants | 977 (9.8) | 2,405 (6.8) | 10.73 |
| ACE inhibitors | 3,682 (36.8) | 13,762 (39.0) | 4.50 |
| Angiotensin II receptor blockers | 4,213 (42.2) | 15,633 (44.3) | 4.39 |
| Beta blockers | 5,150 (51.5) | 20,596 (58.4) | 13.84 |
| Loop diuretics | 2,487 (24.9) | 9,474 (26.9) | 4.52 |
| Other diuretics | 1,911 (19.1) | 8,041 (22.8) | 9.05 |
| Anti thrombotic agents | 2,722 (27.2) | 12,863 (36.5) | 19.93 |
| Calcium channel blockers | 3,182 (31.8) | 11,902 (33.8) | 4.07 |
| Corticosteroids | 63 (0.6) | 243 (0.7) | 0.72 |
| Bisphosphonates | 96 (1.0) | 444 (1.3) | 2.85 |
| Statins | 4,621 (46.2) | 19,200 (54.4) | 16.47 |
| Fibrates | 187 (1.9) | 577 (1.6) | 1.79 |
| Systemic corticosteroids | 1,170 (11.7) | 3,804 (10.8) | 2.91 |
| Tetracyclines | 351 (3.5) | 978 (2.8) | 4.24 |
| Tamoxifen | 54 (0.5) | 114 (0.3) | 3.31 |
| Methotrexate | 149 (1.5) | 399 (1.1) | 3.16 |
| Amiodaron | 59 (0.6) | 548 (1.6) | 9.37 |
| Obeticholic acid | 0 (0.0) | 1 (0.0) | 0.75 |
| Glucose-lowering drugs, n (%) |  |  |  |
| Metformin | 7,745 (77.5) | 27,559 (78.2) | 1.56 |
| Alpha glucosidase inhibitors | 48 (0.5) | 126 (0.4) | 1.90 |
| Sulfonylureas | 908 (9.1) | 3,469 (9.8) | 2.57 |
| DPP-4 inhibitors | 4,322 (43.3) | 15,257 (43.3) | 0.03 |
| Insulin and analogues | 4,307 (43.1) | 8,516 (24.1) | 40.94 |
| Total dispensations of DPP-4 inhibitors and sulfonylureas, mean (SD) | 1.7 (2.2) | 1.7 (2.3) | 3.05 |
| Total dispensations of insulin and analogs, mean (SD) | 3.0 (5.0) | 1.5 (3.6) | 34.20 |
| Health-seeking behaviour, n (%) |  |  |  |
| Influenza vaccination | 3,692 (36.9) | 15,087 (42.8) | 11.95 |
| Breast cancer screening | 416 (4.2) | 941 (2.7) | 8.23 |
| Neoplasm screening for men | 1,075 (10.8) | 5,676 (16.1) | 15.71 |
| Colonoscopy | 62 (0.6) | 253 (0.7) | 1.19 |
| Neoplasm screening for women | 590 (5.9) | 1,908 (5.4) | 2.14 |
| Skin cancer screening | 998 (10.0) | 3,196 (9.1) | 3.15 |
| Cohort entry year, n (%) |  |  |  |
| 2013 | 435 (4.4) | 417 (1.2) | 19.42 |
| 2014 | 392 (3.9) | 1,025 (2.9) | 5.60 |
| 2015 | 596 (6.0) | 1,244 (3.5) | 11.48 |
| 2016 | 659 (6.6) | 2,209 (6.3) | 1.35 |
| 2017 | 638 (6.4) | 2,549 (7.2) | 3.35 |
| 2018 | 784 (7.8) | 2,614 (7.4) | 1.63 |
| 2019 | 1,118 (11.2) | 3,217 (9.1) | 6.84 |
| 2020 | 1,214 (12.1) | 3,504 (9.9) | 7.06 |
| 2021 | 1,557 (15.6) | 4,965 (14.1) | 4.22 |
| 2022 | 1,262 (12.6) | 6,462 (18.3) | 15.80 |
| 2023 | 1,338 (13.4) | 7,057 (20.0) | 17.83 |
| Hospital admissions, n (%) |  |  |  |
| 0 | 6,236 (62.4) | 22,109 (62.7) | 0.61 |
| 1 | 2,306 (23.1) | 7,702 (21.8) | 2.96 |
| 2 | 859 (8.6) | 3,000 (8.5) | 0.32 |
| ≥ 3 | 592 (5.9) | 2,452 (7.0) | 4.19 |
| Outpatient admissions, n (%) |  |  |  |
| ≥ 1 and ≤ 10 | 1,743 (17.4) | 7,157 (20.3) | 7.30 |
| ≥ 11 and ≤ 20 | 4,858 (48.6) | 18,353 (52.0) | 6.87 |
| ≥ 21 and ≤ 30 | 2,539 (25.4) | 7,721 (21.9) | 8.27 |
| > 30 | 853 (8.5) | 2,032 (5.8) | 10.78 |
| Federal state, n (%) |  |  |  |
| Unknown | 27 (0.3) | 85 (0.2) | 0.58 |
| Schleswig-Holstein | 221 (2.2) | 1,146 (3.2) | 6.37 |
| Hamburg (Hanseatic City) | 114 (1.1) | 386 (1.1) | 0.44 |
| Lower Saxony | 881 (8.8) | 2,836 (8.0) | 2.79 |
| Bremen (Hanseatic City) | 23 (0.2) | 99 (0.3) | 1.00 |
| North Rhine-Westphalia | 2,479 (24.8) | 7,824 (22.2) | 6.18 |
| Hesse | 812 (8.1) | 2,819 (8.0) | 0.48 |
| Rhineland-Palatinate | 427 (4.3) | 1,950 (5.5) | 5.82 |
| Bade-Württemberg | 474 (4.7) | 2,393 (6.8) | 8.77 |
| Bavaria (Free State) | 1,269 (12.7) | 4,841 (13.7) | 3.04 |
| Saarland | 141 (1.4) | 630 (1.8) | 2.99 |
| Berlin | 512 (5.1) | 1,656 (4.7) | 1.98 |
| Brandenburg | 693 (6.9) | 2,152 (6.1) | 3.37 |
| Mecklenburg-Western Pomerania | 493 (4.9) | 1,252 (3.6) | 6.87 |
| Saxony (Free State) | 530 (5.3) | 1,884 (5.3) | 0.17 |
| Saxony-Anhalt | 506 (5.1) | 1,881 (5.3) | 1.22 |
| Thuringia (Free State) | 391 (3.9) | 1,429 (4.1) | 0.71 |

Abbreviations: ACE, angiotensin-converting enzyme; ASD, absolute standardised difference; COPD, chronic obstructive pulmonary disease; DPP-4, dipeptidyl peptidase-4; GLP-1 RA, glucagon-like peptide-1 receptor agonist; MASLD, metabolic dysfunction-associated steatotic liver disease; SD, standard deviation; SGLT-2, sodium-glucose cotransporter-2.

## Table S6. Weighted absolute risks and risk differences for the primary outcome MALO comparing new users of GLP-1 RAs with SGLT-2 inhibitors after propensity score weighting

| **…** | **Absolute risk GLP-1 RAs^a^, % (95% CI)** | **Absolute risk SGLT-2 inhibitors^a^,**  **% (95% CI)** | **RD^a^, % (95% CI)** |
| --- | --- | --- | --- |
| **MALO** | … | … | … |
| 1 year | 0.3 (0.2–0.4) | 0.4 (0.3–0.5) | -0.1 (-0.2 to 0.0) |
| 2 years | 0.8 (0.6–1.0) | 0.8 (0.7–0.9) | 0.0 (-0.2 to 0.2) |
| 3 years | 1.1 (0.9–1.3) | 1.3 (1.1–1.5) | -0.2 (-0.5 to 0.1) |
| 4 years | 1.6 (1.3–1.8) | 1.8 (1.6–2.0) | -0.2 (-0.6 to 0.1) |
| 5 years | 2.2 (1.8–2.6) | 2.3 (2.1–2.6) | -0.1 (-0.6 to 0.3) |
| 6 years | 3.1 (2.6–3.6) | 3.0 (2.6–3.3) | 0.1 (-0.5 to 0.7) |
| 7 years | 3.4 (2.8–3.9) | 3.6 (3.2–4.0) | -0.3 (-0.9 to 0.4) |
| 8 years | 4.1 (3.5–4.8) | 4.3 (3.8–4.8) | -0.2 (-1.0 to 0.6) |
| 9 years | 4.6 (3.9–5.3) | 4.9 (4.3–5.5) | -0.3 (-1.2 to 0.7) |
| 10 years | 5.0 (4.2–5.8) | 5.6 (4.8–6.3) | -0.5 (-1.7 to 0.6) |

^a^ weighted using matching weights

Abbreviations: CI, confidence interval; GLP-1 RA, glucagon-like peptide-1 receptor agonist; MALO, major adverse liver outcomes; RD, risk difference; SGLT-2, sodium-glucose cotransporter-2.

## Table S7. Results for the primary outcome and secondary outcomes comparing new users of GLP-1 RAs with SGLT-2 inhibitors before propensity score weighting (Intention-to-treat exposure definition)

| Outcome | People, N | Events | Person-years | IR^a^ (95% CI) | HR (95% CI) |
| --- | --- | --- | --- | --- | --- |
| Primary outcome |  |  |  |  |  |
| MALO |  |  |  |  |  |
| GLP-1 RAs | 9,993 | 226 | 49,384 | 4.6 (4.0–5.2) | 0.77 (0.66–0.89) |
| SGLT-2 inhibitors | 35,263 | 881 | 148,685 | 5.9 (5.5–6.3) | Reference |
| Secondary outcomes |  |  |  |  |  |
| Liver cirrhosis |  |  |  |  |  |
| GLP-1 RAs | 9,993 | 133 | 49,414 | 2.7 (2.3–3.2) | 0.90 (0.74–1.10) |
| SGLT-2 inhibitors | 35,263 | 435 | 148,953 | 2.9 (2.7–3.2) | Reference |
| Hepatic decompensation |  |  |  |  |  |
| GLP-1 RAs | 9,993 | 215 | 49,412 | 4.4 (3.8–5.0) | 0.77 (0.66–0.89) |
| SGLT-2 inhibitors | 35,263 | 838 | 148,770 | 5.6 (5.3–6.0) | Reference |
| Liver transplantation |  |  |  |  |  |
| GLP-1 RAs | 9,993 | 0 | 49,698 | - | - |
| SGLT-2 inhibitors | 35,263 | 2 | 149,778 | 0.0 (0.0–0.1) | Reference |
| HCC |  |  |  |  |  |
| GLP-1 RAs | 9,993 | 21 | 49,663 | 0.4 (0.3–0.6) | 0.62 (0.39–1.00) |
| SGLT-2 inhibitors | 35,263 | 96 | 149,664 | 0.6 (0.5–0.8) | Reference |
| All-cause mortality |  |  |  |  |  |
| GLP-1 RAs | 9,993 | 1,002 | 49,698 | 20.2 (18.9–21.5) | 0.71 (0.66–0.76) |
| SGLT-2 inhibitors | 35,263 | 4,162 | 149,782 | 27.8 (26.9–28.6) | Reference |

^a^ per 1,000 person-years

Abbreviations: CI, confidence interval; GLP-1 RA, glucagon-like peptide-1 receptor agonist; HCC, hepatocellular carcinoma; HR, hazard ratio; IR, incidence rate; MALO, major adverse liver outcomes; SGLT-2, sodium-glucose cotransporter-2.

## Table S8. Results for the primary outcome and secondary outcomes comparing new users of GLP-1 RAs with SGLT-2 inhibitors after propensity score weighting (Intention-to-treat exposure definition, censoring upon tirzepatide initiation)

| Outcome | People, N | Events | Person-years | Weighted IR^a,b^ (95% CI) | Weighted HR^b^ (95% CI) |
| --- | --- | --- | --- | --- | --- |
| Primary outcome |  |  |  |  |  |
| MALO |  |  |  |  |  |
| GLP-1 RAs | 9,484 | 215 | 45,482 | 4.7 (4.1–5.4) | 0.92 (0.78–1.08) |
| SGLT-2 inhibitors | 9,416 | 235 | 45,485 | 5.2 (4.5–5.9) | Reference |
| Secondary outcomes |  |  |  |  |  |
| Liver cirrhosis |  |  |  |  |  |
| GLP-1 RAs | 9,484 | 124 | 45,521 | 2.7 (2.3–3.3) | 0.89 (0.72–1.10) |
| SGLT-2 inhibitors | 9,416 | 140 | 45,510 | 3.1 (2.6–3.6) | Reference |
| Hepatic decompensation |  |  |  |  |  |
| GLP-1 RAs | 9,484 | 205 | 45,503 | 4.5 (3.9–5.2) | 0.91 (0.77–1.08) |
| SGLT-2 inhibitors | 9,416 | 225 | 45,511 | 4.9 (4.3–5.6) | Reference |
| Liver transplantation |  |  |  |  |  |
| GLP-1 RAs | 9,484 | 0 | - | - | - |
| SGLT-2 inhibitors | 9,416 | 0 | - | - | Reference |
| HCC |  |  |  |  |  |
| GLP-1 RAs | 9,484 | 20 | 45,745 | 0.4 (0.3–0.7) | 0.76 (0.45–1.30) |
| SGLT-2 inhibitors | 9,416 | 26 | 45,800 | 0.6 (0.4–0.8) | Reference |
| All-cause mortality |  |  |  |  |  |
| GLP-1 RAs | 9,484 | 949 | 45,773 | 20.7 (19.4–22.1) | 1.04 (0.96–1.12) |
| SGLT-2 inhibitors | 9,416 | 916 | 45,839 | 20.0 (18.7–21.3) | Reference |

^a^ per 1,000 person-years

^b^ weighted using matching weights

Abbreviations: CI, confidence interval; GLP-1 RA, glucagon-like peptide-1 receptor agonist; HCC, hepatocellular carcinoma; HR, hazard ratio; IR, incidence rate; MALO, major adverse liver outcomes; SGLT-2, sodium-glucose cotransporter-2.

## Table S9. Results for the primary outcome and secondary outcomes comparing new users of GLP-1 RAs with SGLT-2 inhibitors after propensity score weighting (on-treatment exposure definition)

| Outcome | People, N | Events | Person-years | Weighted IR^a,b^ (95%CI) | Weighted HR^b^ (95%CI) |
| --- | --- | --- | --- | --- | --- |
| Primary outcome |  |  |  |  |  |
| MALO |  |  |  |  |  |
| GLP-1 RAs | 9,484 | 62 | 18,763 | 3.3 (2.5–4.2) | 0.78 (0.58–1.03) |
| SGLT-2 inhibitors | 9,416 | 77 | 17,997 | 4.3 (3.4–5.4) | Reference |
| Secondary outcomes |  |  |  |  |  |
| Liver cirrhosis |  |  |  |  |  |
| GLP-1 RAs | 9,484 | 33 | 18,752 | 1.7 (1.2–2.4) | 0.84 (0.56–1.25) |
| SGLT-2 inhibitors | 9,416 | 37 | 18,012 | 2.1 (1.5–2.9) | Reference |
| Hepatic decompensation |  |  |  |  |  |
| GLP-1 RAs | 9,484 | 60 | 18,766 | 3.2 (2.4–4.1) | 0.77 (0.58–1.04) |
| SGLT-2 inhibitors | 9,416 | 75 | 18,004 | 4.2 (3.3–5.2) | Reference |
| Liver transplantation |  |  |  |  |  |
| GLP-1 RAs | 9,484 | 0 | - | - | - |
| SGLT-2 inhibitors | 9,416 | 0 | - | - |  |
| HCC |  |  |  |  | Reference |
| GLP-1 RAs | 9,484 | 6 | 18,791 | 0.3 (0.1–0.7) | 1.03 (0.41–2.58) |
| SGLT-2 inhibitors | 9,416 | 6 | 18,049 | 0.3 (0.1–0.7) | Reference |
| All-cause mortality |  |  |  |  |  |
| GLP-1 RAs | 9,484 | 240 | 18,794 | 12.8 (11.2–14.5) | 0.87 (0.75–1.00) |
| SGLT-2 inhibitors | 9,416 | 268 | 18,057 | 14.9 (13.1–16.7) | Reference |

^a^ per 1,000 person-years

^b^ weighted using matching weights

Abbreviations: CI, confidence interval; GLP-1 RA, glucagon-like peptide-1 receptor agonist; HCC, hepatocellular carcinoma; HR, hazard ratio; IR, incidence rate; MALO, major adverse liver outcomes; SGLT-2, sodium-glucose cotransporter-2.

## Table S10. Censoring reasons and their relative proportions for the on-treatment exposure definition for both treatment groups of new users of GLP-1 RAs and SGLT-2 inhibitors

| **Reason** | **GLP-1 RAs** | **SGLT-2 inhibitors** |
| --- | --- | --- |
| End of Study | 17.7% | 23.1% |
| Drug discontinuation | 53.9% | 54.4% |
| Death | 2.3% | 2.5% |
| Switch to/ add comparator drug | 25.0% | 18.8% |
| End of insurance | 1.1% | 1.1% |

Abbreviations: GLP-1 RA, glucagon-like peptide-1 receptor agonist; SGLT-2, sodium-glucose cotransporter-2.

## Table S11. Results for the primary outcome and secondary outcomes comparing new users of GLP-1 RAs with SGLT-2 inhibitors after propensity score weighting (on-treatment exposure definition, censoring upon tirzepatide initiation)

| Outcome | People, N | Events | Person-years | Weighted IR^a,b^ (95%CI) | Weighted HR^b^ (95%CI) |
| --- | --- | --- | --- | --- | --- |
| Primary outcome |  |  |  |  |  |
| MALO |  |  |  |  |  |
| GLP-1 RAs | 9,484 | 62 | 18,710 | 3.3 (2.5–4.3) | 0.78 (0.58–1.04) |
| SGLT-2 inhibitors | 9,416 | 77 | 17,968 | 4.3 (3.4–5.4) | Reference |
| Secondary outcomes |  |  |  |  |  |
| Liver cirrhosis |  |  |  |  |  |
| GLP-1 RAs | 9,484 | 33 | 18,699 | 1.7 (1.2–2.5) | 0.84 (0.56–1.26) |
| SGLT-2 inhibitors | 9,416 | 37 | 17,983 | 2.1 (1.5–2.9) | Reference |
| Hepatic decompensation |  |  |  |  |  |
| GLP-1 RAs | 9,484 | 60 | 18,712 | 3.2 (2.5–4.1) | 0.78 (0.58–1.04) |
| SGLT-2 inhibitors | 9,416 | 75 | 17,974 | 4.2 (3.3–5.2) | Reference |
| Liver transplantation |  |  |  |  |  |
| GLP-1 RAs | 9,484 | 0 | - | - | - |
| SGLT-2 inhibitors | 9,416 | 0 | - | - |  |
| HCC |  |  |  |  | Reference |
| GLP-1 RAs | 9,484 | 6 | 18,737 | 0.3 (0.1–0.7) | 1.03 (0.41–2.58) |
| SGLT-2 inhibitors | 9,416 | 6 | 18,020 | 0.3 (0.1–0.7) | Reference |
| All-cause mortality |  |  |  |  |  |
| GLP-1 RAs | 9,484 | 240 | 18,740 | 12.8 (11.3–14.6) | 0.87 (0.75–1.00) |
| SGLT-2 inhibitors | 9,416 | 268 | 18,028 | 14.9 (13.1–16.8) | Reference |

^a^ per 1,000 person-years

^b^ weighted using matching weights

Abbreviations: CI, confidence interval; GLP-1 RA, glucagon-like peptide-1 receptor agonist; HCC, hepatocellular carcinoma; HR, hazard ratio; IR, incidence rate; MALO, major adverse liver outcomes; SGLT-2, sodium-glucose cotransporter-2.

## Table S12. Censoring reasons and their relative proportions for the on-treatment exposure definition (including censoring upon tirzepatide initiation) for both treatment groups of new users of GLP-1 RAs and SGLT-2 inhibitors

| **Reason** | **GLP-1 RAs** | **SGLT-2 inhibitors** |
| --- | --- | --- |
| End of Study | 17.2% | 22.5% |
| Drug discontinuation | 52.1% | 54.2% |
| Death | 2.3% | 2.5% |
| Switch to/ add comparator drug | 25.0% | 18.8% |
| End of insurance | 1.1% | 1.1% |
| Tirzepatide initiation | 2.4% | 0.8% |

Abbreviations: GLP-1 RA, glucagon-like peptide-1 receptor agonist; SGLT-2, sodium-glucose cotransporter-2.

## Table S13. Results for the primary outcome and secondary outcomes comparing new users of GLP-1 RAs with SGLT-2 inhibitors after propensity score weighting (Standardised morbidity ratio weighting)

| Outcome | People, N | Events | Person-years | Weighted IR^a,b^ (95% CI) | Weighted HR^b^ (95% CI) |
| --- | --- | --- | --- | --- | --- |
| Primary outcome |  |  |  |  |  |
| MALO |  |  |  |  |  |
| GLP-1 RAs | 9,993 | 226 | 49,384 | 4.6 (4.0–5.2) | 0.88 (0.73–1.05) |
| SGLT-2 inhibitors | 10,298 | 274 | 52,119 | 5.3 (4.7–5.9) | Reference |
| Secondary outcomes |  |  |  |  |  |
| Liver cirrhosis |  |  |  |  |  |
| GLP-1 RAs | 9,993 | 133 | 49,414 | 2.7 (2.3–3.2) | 0.79 (0.62–1.02) |
| SGLT-2 inhibitors | 10,298 | 179 | 52,113 | 3.4 (2.9–4.0) | Reference |
| Hepatic decompensation |  |  |  |  |  |
| GLP-1 RAs | 9,993 | 215 | 49,412 | 4.4 (3.8–5.0) | 0.87 (0.72–1.04) |
| SGLT-2 inhibitors | 10,298 | 264 | 52,146 | 5.1 (4.5–5.7) | Reference |
| Liver transplantation |  |  |  |  |  |
| GLP-1 RAs | 9,993 | 0 | - | - | - |
| SGLT-2 inhibitors | 10,298 | 0 | - | - | Reference |
| HCC |  |  |  |  |  |
| GLP-1 RAs | 9,993 | 21 | 49,663 | 0.4 (0.3–0.6) | 0.56 (0.28–1.15) |
| SGLT-2 inhibitors | 10,298 | 39 | 52,558 | 0.7 (0.5–1.0) | Reference |
| All-cause mortality |  |  |  |  |  |
| GLP-1 RAs | 9,993 | 1,002 | 49,698 | 20.2 (18.9–21.5) | 1.07 (0.99–1.16) |
| SGLT-2 inhibitors | 10,298 | 1,000 | 52,599 | 19.0 (17.8–20.2) | Reference |

^a^ per 1,000 person-years

^b^ weighted using standardised morbidity ratio weighting

Abbreviations: CI, confidence interval; GLP-1 RA, glucagon-like peptide-1 receptor agonist; HCC, hepatocellular carcinoma; HR, hazard ratio; IR, incidence rate; MALO, major adverse liver outcomes; SGLT-2, sodium-glucose cotransporter-2.

## Table S14. Results for the primary outcome and secondary outcomes comparing new users of GLP-1 RAs with SGLT-2 inhibitors after propensity score weighting (Inverse probability of treatment weighting)

| Outcome | People, N | Events | Person-years | Weighted IR^a,b^ (95% CI) | Weighted HR^b^ (95% CI) |
| --- | --- | --- | --- | --- | --- |
| Primary outcome |  |  |  |  |  |
| MALO |  |  |  |  |  |
| GLP-1 RAs | 43,828 | 1,040 | 198,868 | 5.2 (4.9–5.6) | 0.91 (0.75–1.10) |
| SGLT-2 inhibitors | 45,561 | 1,155 | 200,804 | 5.8 (5.4–6.1) | Reference |
| Secondary outcomes |  |  |  |  |  |
| Liver cirrhosis |  |  |  |  |  |
| GLP-1 RAs | 43,828 | 493 | 199,165 | 2.5 (2.3–2.7) | 0.81 (0.64–1.02) |
| SGLT-2 inhibitors | 45,561 | 614 | 201,066 | 3.1 (2.8–3.3) | Reference |
| Hepatic decompensation |  |  |  |  |  |
| GLP-1 RAs | 43,828 | 1,004 | 198,951 | 5.0 (4.7–5.4) | 0.92 (0.76–1.11) |
| SGLT-2 inhibitors | 45,561 | 1,102 | 200,917 | 5.5 (5.2–5.8) | Reference |
| Liver transplantation |  |  |  |  |  |
| GLP-1 RAs | 43,828 | 0 | - | - | - |
| SGLT-2 inhibitors | 45,561 | 2 | 202,377 | 0.0 (0.0–0.0) | Reference |
| HCC |  |  |  |  |  |
| GLP-1 RAs | 43,828 | 84 | 200,011 | 0.4 (0.3–0.5) | 0.61 (0.35–1.06) |
| SGLT-2 inhibitors | 45,561 | 135 | 202,221 | 0.7 (0.6–0.8) | Reference |
| All-cause mortality |  |  |  |  |  |
| GLP-1 RAs | 43,828 | 4,939 | 200,117 | 24.7 (24.0–25.4) | 0.96 (0.88–1.06) |
| SGLT-2 inhibitors | 45,561 | 5,162 | 202,381 | 25.5 (24.8–26.2) | Reference |

^a^ per 1,000 person-years

^b^ weighted using inverse probability of treatment weighting

Abbreviations: CI, confidence interval; GLP-1 RA, glucagon-like peptide-1 receptor agonist; HCC, hepatocellular carcinoma; HR, hazard ratio; IR, incidence rate; MALO, major adverse liver outcomes; SGLT-2, sodium-glucose cotransporter-2.

## Table S15. Results for the primary outcome and secondary outcomes comparing new users of GLP-1 RAs with SGLT-2 inhibitors after propensity score weighting (Restricting outcomes to inpatient diagnoses in primary position)

| Outcome | People, N | Events | Person-years | Weighted IR^a,b^ (95% CI) | Weighted HR^b^ (95% CI) |
| --- | --- | --- | --- | --- | --- |
| Primary outcome |  |  |  |  |  |
| MALO |  |  |  |  |  |
| GLP-1 RAs | 9,484 | 52 | 46,170 | 1.1 (0.8–1.5) | 0.77 (0.56–1.07) |
| SGLT-2 inhibitors | 9,416 | 68 | 46,005 | 1.5 (1.1–1.9) | Reference |
| Secondary outcomes |  |  |  |  |  |
| Liver cirrhosis |  |  |  |  |  |
| GLP-1 RAs | 9,484 | 42 | 46,186 | 0.9 (0.7–1.2) | 0.74 (0.51–1.06) |
| SGLT-2 inhibitors | 9,416 | 57 | 46,009 | 1.2 (0.9–1.6) | Reference |
| Hepatic decompensation |  |  |  |  |  |
| GLP-1 RAs | 9,484 | 37 | 46,195 | 0.8 (0.6–1.1) | 0.71 (0.48–1.04) |
| SGLT-2 inhibitors | 9,416 | 51 | 46,037 | 1.1 (0.8–1.5) | Reference |
| Liver transplantation |  |  |  |  |  |
| GLP-1 RAs | 9,484 | 0 | - | - | - |
| SGLT-2 inhibitors | 9,416 | 0 | - | - | Reference |
| HCC |  |  |  |  |  |
| GLP-1 RAs | 9,484 | 17 | 46,248 | 0.4 (0.2–0.6) | 0.74 (0.42–1.30) |
| SGLT-2 inhibitors | 9,416 | 23 | 46,089 | 0.5 (0.3–0.7) | Reference |
| All-cause mortality |  |  |  |  |  |
| GLP-1 RAs | 9,484 | 956 | 46,273 | 20.7 (19.4–22.0) | 1.04 (0.96–1.12) |
| SGLT-2 inhibitors | 9,416 | 919 | 46,126 | 19.9 (18.7–21.3) | Reference |

^a^ per 1,000 person-years

^b^ weighted using matching weights

Abbreviations: CI, confidence interval; GLP-1 RA, glucagon-like peptide-1 receptor agonist; HCC, hepatocellular carcinoma; HR, hazard ratio; IR, incidence rate; MALO, major adverse liver outcomes; SGLT-2, sodium-glucose cotransporter-2.

# Supplementary Figures

## Figure S1. Propensity score distribution before weighting on the propensity score (A) and after weighting using matching weights (B) for new users of GLP-1 RAs and SGLT-2 inhibitors

Abbreviations: GLP-1 RA, glucagon-like peptide-1 receptor agonist; SGLT-2, sodium-glucose cotransporter-2.

## Figure S2. Weighted cumulative incidence curves of incident liver cirrhosis among new users of GLP-1 RAs and SGLT-2 inhibitors

Abbreviations: GLP-1 RA, glucagon-like peptide-1 receptor agonist; SGLT-2, sodium-glucose cotransporter-2.

## Figure S3. Weighted cumulative incidence curves of incident hepatic decompensation among new users of GLP-1 RAs and SGLT-2 inhibitors

Abbreviations: GLP-1 RA, glucagon-like peptide-1 receptor agonist; SGLT-2, sodium-glucose cotransporter-2.

## Figure S4. Weighted cumulative incidence curves of incident hepatocellular carcinoma among new users of GLP-1 RAs and SGLT-2 inhibitors

Abbreviations: GLP-1 RA, glucagon-like peptide-1 receptor agonist; SGLT-2, sodium-glucose cotransporter-2.

## Figure S5. Weighted cumulative incidence curves of all-cause mortality among new users of GLP-1 RAs and SGLT-2 inhibitors

Abbreviations: GLP-1 RA, glucagon-like peptide-1 receptor agonist; SGLT-2, sodium-glucose cotransporter-2.

## Figure S6. Primary analysis and each subgroup analysis for new users of GLP-1 RAs compared with SGLT-2 inhibitors for the primary composite outcome major adverse liver outcomes

Cardiovascular disease (CVD): at least one of the following conditions within one year up to the cohort index date: I60x-I64x, I20x, I21x, I22x, I24x, I25x, I739, I702, I50x, I130, I110, I132, 5-361x, 5-362x,5-363x, 8-836x, 8-837x, 8-84.x; Kidney disease: at least one of the conditions within one year up to the index date: renal failure and/or diabetic nephropathy.
Abbreviations: CI, confidence interval; GLP-1 RA, glucagon-like peptide-1 receptor agonist; SGLT-2, sodium-glucose cotransporter-2.

REFERENCES

1.	Reitzle L, Ihle P, Heidemann C, Paprott R, Koster I, Schmidt C. [Algorithm for the Classification of Type 1 and Type 2 Diabetes Mellitus for the Analysis of Routine Data]. *Gesundheitswesen.* 2023;85(S 02):S119-S126.

2.	Reitzle L, Schmidt C, Du Y, et al. [Estimating prevalent microvascular complications of diabetes mellitus in Germany. Analysis of statutory health insurance data in 2012 and 2013]. *Bundesgesundheitsblatt Gesundheitsforschung Gesundheitsschutz.* 2020;63(10):1219-1230.

3.	Reitzle L, Koster I, Tuncer O, Schmidt C, Meyer I. [Development and Internal Validation of Case Definitions for Prevalence Estimation of Microvascular Complications of Diabetes in Routine Data]. *Gesundheitswesen.* 2024;86(S 03):S196-S204.
